# The Expression and Function of Tubulin Isotypes in *Caenorhabditis elegans*

**DOI:** 10.3389/fcell.2022.860065

**Published:** 2022-03-24

**Authors:** Yu-Ming Lu, Chaogu Zheng

**Affiliations:** School of Biological Sciences, University of Hong Kong, Hong Kong SAR, China

**Keywords:** microtubules, *C. elegans*, scrna-seq, tubulin code, tubulin PTM, tubulin (poly)glutamylation, touch receptor neurons, axonemal microtubules

## Abstract

Microtubules, made from the polymerization of the highly conserved α/β-tubulin heterodimers, serve as important components of the cytoskeleton in all eukaryotic cells. The existence of multiple tubulin isotypes in metazoan genomes and a dazzling variety of tubulin posttranslational modifications (PTMs) prompted the “tubulin code” hypothesis, which proposed that microtubule structure and functions are determined by the tubulin composition and PTMs. Evidence for the tubulin code has emerged from studies in several organisms with the characterization of specific tubulins for their expression and functions. The studies of tubulin PTMs are accelerated by the discovery of the enzymes that add or remove the PTMs. In tubulin research, the use of simple organisms, such as *Caenorhabditis elegans*, has been instrumental for understanding the expression and functional specialization of tubulin isotypes and the effects of their PTMs. In this review, we summarize the current understanding of the expression patterns and cellular functions of the nine α-tubulin and six β-tubulin isotypes. Expression studies are greatly facilitated by the CRISPR/Cas9-mediated endogenous GFP knock-in reporters and the organism-wide single cell transcriptomic studies. Meanwhile, functional studies benefit from the ease of genetic manipulation and precise gene replacement in *C. elegans*. These studies identified both ubiquitously expressed tubulin isotypes and tissue-specific isotypes. The isotypes showed functional redundancy, as well as functional specificity, which is likely caused by the subtle differences in their amino acid sequences. Many of these differences concentrate at the C-terminal tails that are subjected to several PTMs. Indeed, tubulin PTM, such as polyglutamylation, is shown to modulate microtubule organization and properties in both ciliated and non-ciliated neurons. Overall, studies from *C. elegans* support the distinct expression and function patterns of tubulin isotypes and the importance of their PTMs and offer the promise of cracking the tubulin code at the whole-genome and the whole-organism level.

## Introduction

Microtubules perform pivotal roles in numerous cellular activities, including cell division, cell shape changes, intracellular trafficking, sperm motility, and neuronal sensation. In dividing cells, kinetochore microtubules attach chromosomes to the spindle pole, allowing the separation of chromosomes into daughter cells, while astral microtubules radiate out from spindle poles toward the cell cortex to help orient the spindles and control the plane of cell division (Prosser and Pelletier 2017). In the nervous system, microtubules provide mechanical support for axonal outgrowth and serve as transport highways for organelles and vesicles from the cell bodies to axons and dendrites (Matamoros and Baas 2016). Two other specialized organelles, the motile flagella and non-motile cilia, also rely heavily on microtubules, as the microtubule-based axoneme structures control the locomotion of flagellated sperms and how ciliated neurons sense the environment (Guichard et al., 2021; Gadadhar et al., 2022).

Compared to their versatile functions, microtubules have a relatively simple and rigid structure in the shape of 25-nm-wide hollow tubes. They generally harbor 13 linear protofilaments formed from evolutionarily conserved tubulin dimers. After the initiation of *de novo* microtubule assembly (“microtubule nucleation”) by the γ-tubulin ring complex of the microtubule-organizing center (Consolati et al., 2020; Liu et al., 2021), tubulin heterodimers composed of one α- and one β-tubulin are recruited to elongate protofilaments at the plus end. Through incorporating and removing tubulin dimers, microtubules switch between the states of polymerization and depolymerization, a process called dynamic instability. Microtubule dynamics is required for spindle assembly and function in dividing cells (Wittmann et al., 2001) and growth cone navigation during axonal pathfinding of growing or regenerating neurons (Gudimchuk and McIntosh 2021; Sanchez-Huertas and Herrera 2021).

The simplicity of microtubule structure and composition makes it intriguing to unravel how the behavior of the multifunctional microtubules are precisely controlled. Previous studies identified microtubule-associated proteins (MAPs) as important regulators of microtubule dynamics and functions. For example, molecular motors walk along microtubules to transport cargoes and generate movement in cilia (Howard 1997; Webb et al., 2020); microtubule-severing enzymes, such as katanin, can rapidly reorganize microtubules by cutting them into shorter fragments during cell division (Sharp and Ross 2012); MAP2 and tau, on the other hand, promote microtubule assembly and protect microtubules from severing in the nervous system (Qiang et al., 2006; Barbier et al., 2019). In addition to MAPs, tubulins themselves are also important determinants of the properties of microtubules.

Eukaryotes contain multiple tubulin genes, called tubulin isotypes. For instance, the human genome has nine α-, ten β-, and two γ-tubulin isotypes (HGCN “tubulins” gene group). These isotypes, as building blocks of microtubule, may possess specific properties that dictate the structure and function of the entire microtubule polymer. One prominent example is the human β1-tubulin (TUBB1) whose mutation leads to disorganized microtubules of blood platelets and thus congenital macrothrombocytopenia (Kunishima et al., 2009), even though six other β-tubulin isotypes are simultaneously abundant in the cells (Cuenca-Zamora et al., 2019). Given that tubulin isotypes generally bear more than 95% identity in amino acid sequences, the distinct functions of the isotypes likely originate from their subtle sequence variations. A region where significant sequence divergence was found is the carboxy-terminal tail, which protrudes from microtubule backbones and remains highly accessible to MAPs. The C-terminal tail is also subjected to a range of tubulin post translational modifications (PTMs) that regulate microtubule dynamics and MAP binding (Magiera and Janke 2014), and tubulin isotypes with varying C-terminal tails may have different susceptibility to PTMs. Therefore, one could hypothesize that the incorporation of specific tubulin isotypes into the microtubules may regulate microtubule properties by controlling the accessibility to certain MAPs.

Altogether, the diversity of tubulin isotypes and the complexity of tubulin PTMs gave rise to the concept of “tubulin code,” which proposes that the structural and functional properties of microtubules are precisely controlled by the tubulin isotype composition and their PTMs (Janke and Magiera 2020; Roll-Mecak 2020). Evidence for such code is starting to emerge from recent studies, although more work needs to be done to address the intricate regulatory mechanisms of microtubule behaviors. In this paper, we review the tubulin studies in *Caenorhabditis elegans*, which is a powerful model organism thanks to its relatively simple anatomy with only ∼1,000 somatic cells, short (3-days) life cycle, and amenability to genetic manipulations (Brenner 1974; Corsi et al., 2015). *C. elegans* also has transparent embryos and bodies, allowing *in vivo* imaging of microtubule dynamics in living animals. Studies on the expression and function of tubulin isotypes, as well as the effects of their PTMs, provide critical insights into how the isotypes exert specific functions in specific tissues at the whole-organism level.

### Expression Patterns of Tubulin Isotypes in *C. elegans*

#### Tissue Specificity of Tubulin Isotypes

**FIGURE 1:**
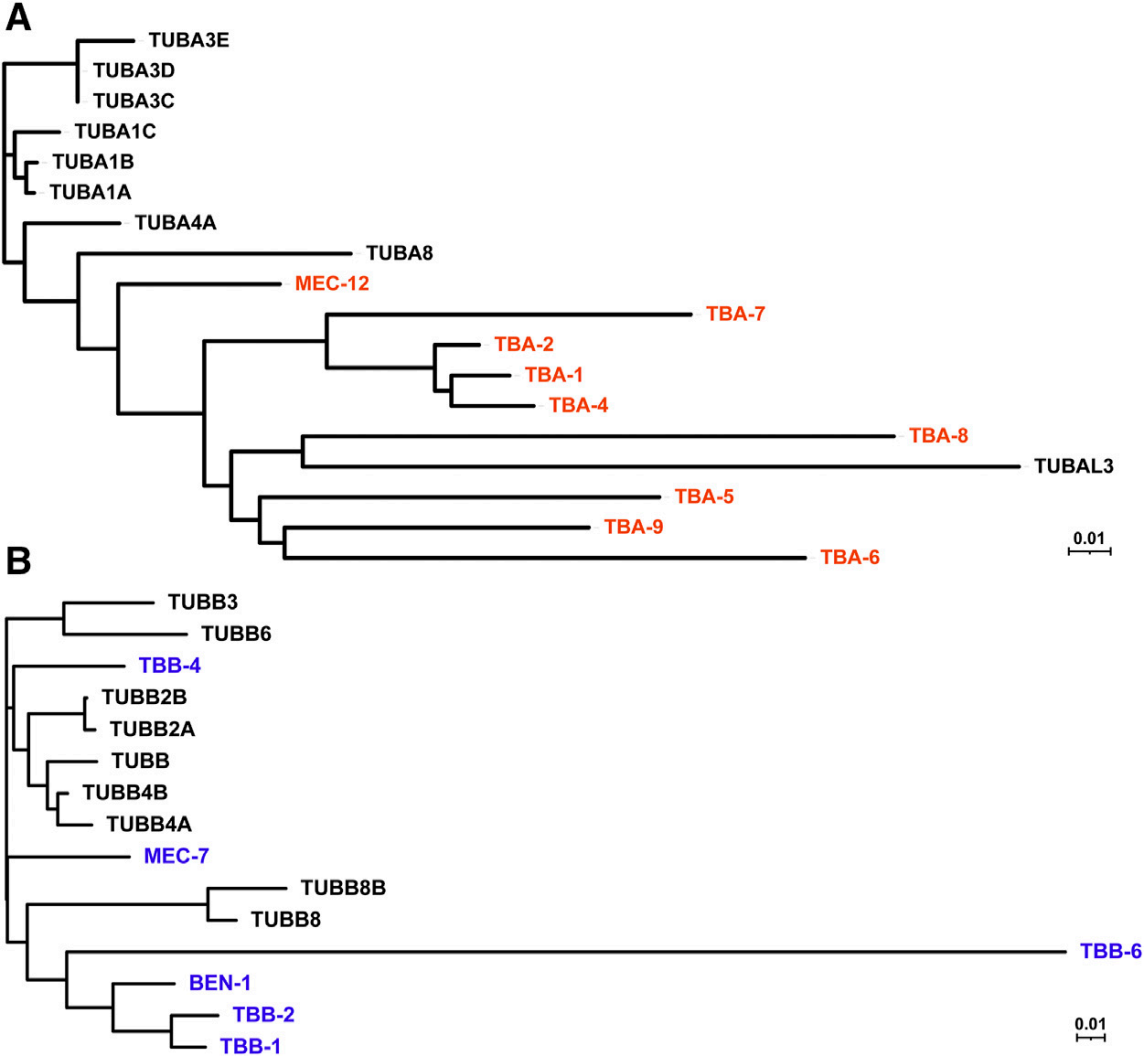
Phylogenetic tree of human and *C. elegans* tubulin isotypes. Neighbor-joining trees were generated by aligning the protein sequences of human and *C. elegans* α-tubulin **(A)** and β-tubulin **(B)** isotypes using ClustalW. *C. elegans* tubulins are in red **(A)** and blue **(B)**, while human tubulins are in black.

*C. elegans* genome contains nine α-, six β-, and one γ-tubulin genes (Figure 1 and Table 1). *C. elegans* has no δ- and ε-tubulins associated with the centrioles (Chang and Stearns 2000). When and where tubulin genes are expressed in *C. elegans* had been studied using a range of methods, including the traditional promoter-reporters, immunofluorescence with tubulin-specific antibodies (Fukushige et al., 1995; Lu et al., 2004), transcriptomic approaches (Baugh et al., 2003; Lockhead et al., 2016), and GFP knock-in at the endogenous tubulin loci (Nishida et al., 2021). Recent single-cell RNA-seq data (Taylor et al., 2021) provide opportunities to understand the combinations of tubulin isotypes expressed in each cell and their relative abundance in a quantitative manner. Based on these expression patterns, *C. elegans* tubulin isotypes can be categorized into 1) mitotic and ubiquitous tubulins, 2) ciliary tubulins, 3) mechanosensory tubulins, 4) other tubulins (Table 1).

**TABLE 1:** Summary of the expression and function of *C. elegans* tubulin isotypes.

Isotype Category	a or b	Gene names	Expression Pattern	Function based on knockout phenotypes
Mitotic and ubiquitous tubulins	α-tubulin	*tba-1*	All tissues	Redundant for spindle formation and dynamics in mitosis and meiosis
		*tba-2*	All tissues	
	β-tubulin	*tbb-1*	All tissues	
		*tbb-2*	All tissues	
Cilliary tubulins	α-tubulin	*tba-5*	ASG, PHA, ASK, ADF, ASE, ASI, and other ciliated neurons; male CEM and ray neurons	Unknown
		*tba-6*	IL2; male CEM and ray neurons	Cilary ultrastructure, intraflagellar transport, and extracellular vesicle release in CEM; male sensory function
		*tba-9*	CEP, ADE, PDE, AWA, AWC, and other ciliated neurons; male CEM and ray neurons	Male sensory function
	β-tubulin	*tbb-4*	Most ciliated neurons	Male sensory function
Mechanosensory tubulins	α-tubulin	*mec-12*	TRNs and most neurons	Mechanosensation
	β-tubulin	*mec-7*	TRNs and a few other neurons	Formation of 15-protofilament microtubule; mechanosensation
Others	α-tubulin	*tba-4*	Epidermis and muscle	Unknown
		*tba-7*	Intestine	Unknown
		*tba-8*	Seam cells	Unknown
	β-tubulin	*ben-1*	Many neurons	Benomyl-sensitive
		*tbb-6*	Pharynx	Unknown

Two α-tubulins (*tba-1* and *tba-2*) and two β-tubulins (*tbb-1* and *tbb-2*) are the four main isotypes expressed in the embryos (Baugh et al., 2003) and virtually all tissues in larva and adults, including *epidermis*, germline, intestine, muscle, and neurons (Nishida et al., 2021). They are incorporated into the mitotic spindle during cell division in the embryos (Honda et al., 2017) and likely contribute to microtubule formation in every cell of the animal as ubiquitously expressed tubulin isotypes.

**FIGURE 2:**
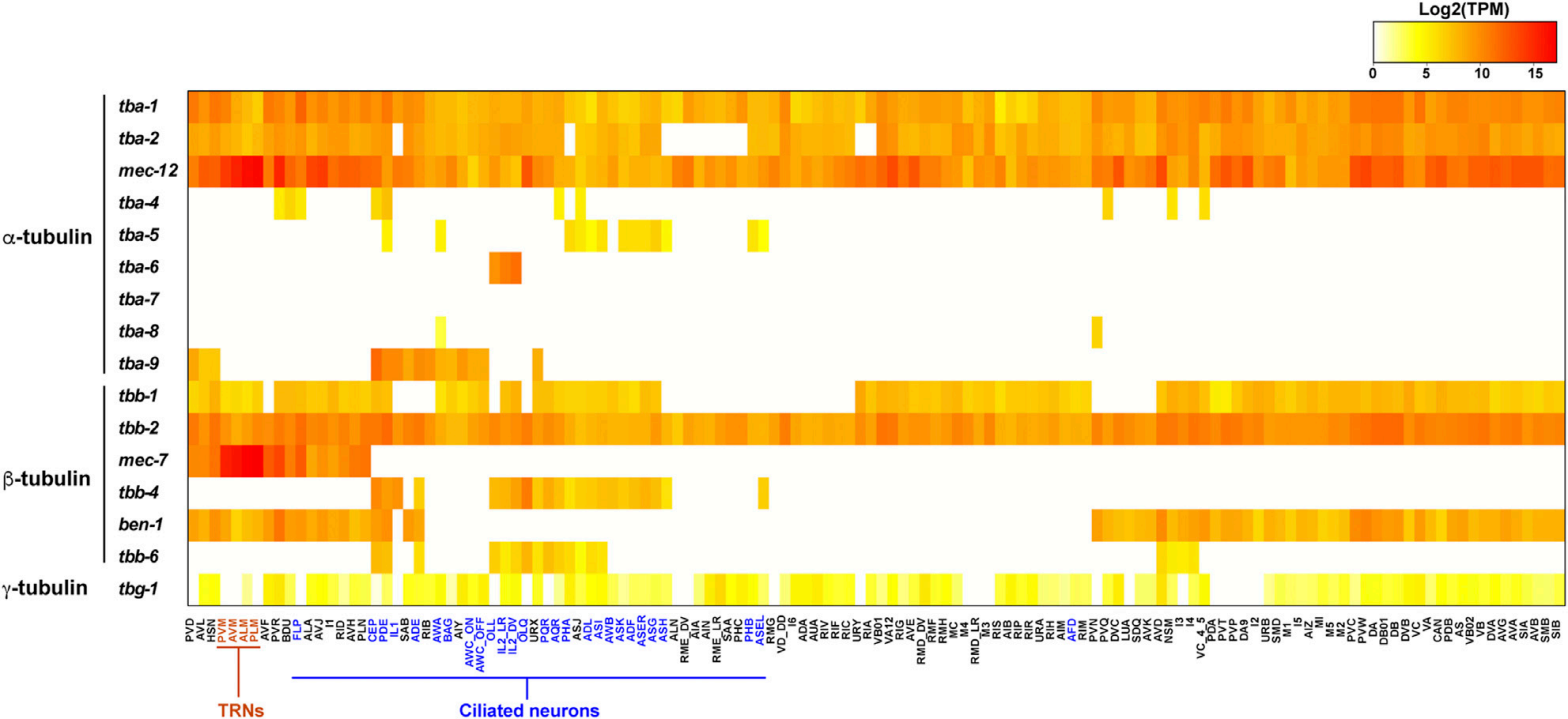
Heatmap for the expression of tubulin isotypes in the *C. elegans* nervous system. Transcript levels (TPM) of tubulin isotypes in each neuron type were extracted from the scRNA-seq data (Taylor et al., 2021) and log2 transformed. TRNs are in red and ciliated neurons are in blue. Raw data can be found in Supplementary Table S1.

Three α-tubulins (*tba-5*, *tba-6*, and *tba-9*) and one β-tubulin (*tbb-4*) are specifically expressed in the ciliated sensory neurons (Hurd et al., 2010; Hao et al., 2011). Among the 302 neurons in *C. elegans* hermaphrodites, 60 of them are ciliated sensory neurons containing axoneme, a microtubule-based structure that forms the core of the cilia. These ciliary isotypes contribute to the formation of axonemal microtubules and cilia functions. Interestingly, among the ciliated sensory neurons, the three α-tubulins showed distinct and largely non-overlapping expression in hermaphrodites. Fluorescent reporter studies found that *tba-5* is expressed in the ADF, ADL, AFD, ASE, ASG, ASH, ASI, ASK, AWA, AWB, and PHA neurons, *tba-6* is expressed in IL2, and *tba-9* is expressed in CEP, ADE, PDE, AWA, and AWC neurons (Hurd et al., 2010; Nishida et al., 2021). Information about each neuron type in *C. elegans* can be found at WormAtlas (https://www.wormatlas.org/neurons/Individual%20Neurons/Neuronframeset.html). scRNA-seq identified their expression in more ciliated neurons but their expression patterns remain mostly non-overlapping (Figure 2). In contrast, the only ciliary β-tubulin (*tbb-4*) is widely expressed in many ciliated neurons (Hao et al., 2011). The partitioned expression of the three ciliary α-tubulin isotypes leads to the hypothesis that the axonemal microtubules may have different properties in different sets of ciliated neurons, thus requiring the expression of distinct isotypes. Whether *tba-5*, *tba-6* and *tba-9* are functionally interchangeable among different ciliated neurons remains to be tested. These ciliary tubulins are also expressed in the male-specific ciliated sensory neurons (Hurd et al., 2010).

One α-tubulin (*mec-12*) and one β-tubulin (*mec-7*) showed highly enriched expression in the mechanosensory neurons, also known as the touch receptor neurons (TRNs) including ALM, AVM, PLM, and PVM (Hamelin et al., 1992; Fukushige et al., 1999). These neurons contain the special 15-protofilament microtubules, while all other neurons in *C. elegans* have 11-protofilament microtubules (Chalfie and Thomson 1982). Mutations in *mec-7* leads to the loss of the 15-protofilament microtubule structure (Savage et al., 1989), suggesting that tubulin isotype can define the structure and organization of microtubules. Both *mec-12* and *mec-7* are required for the mechanosensory functions of TRNs (Chalfie and Au 1989). It is worth noting that *mec-7* is also expressed in a few other neurons outside of the TRNs, and *mec-12* expression is found in many neurons, including motor neurons and interneurons, although their expression in the six TRNs are much higher compared to other expression (Fukushige et al., 1999; Nishida et al., 2021). This expression pattern is supported by scRNA-seq data (Figure 2). Surprisingly, *mec-12* transcripts were found in all neurons at a level comparable to *tba-1* and *tba-2*, suggesting that *mec-12* is potentially a third ubiquitous α-tubulin with specific enrichment in the TRNs.

Other α-tubulin isotypes include *tba-4*, *tba-7*, and *tba-8*, which had very little expression in the nervous system. *tba-4* is expressed in the *epidermis* and muscle, *tba-7* is expressed in the intestine, excretory pore cells, and rectal gland cells, and *tba-8* is expressed specifically in the seam cells of the larva (Hurd 2018; Nishida et al., 2021). Other β-tubulin isotypes include *ben-1* (also known as *tbb-5*), which is the only benomyl-sensitive tubulin whose null mutation renders the animals resistant to the toxic fungicide and is expressed in many neurons (Driscoll et al., 1989; Hurd 2018), and *tbb-6*, which has expression only in the pharynx (Munkacsy et al., 2016). The only *C. elegans* γ-tubulin isotype (*tbg-1*), involved in the initiation of microtubule assembly, is expressed in the germline, embryos, and likely all somatic cells (Strome et al., 2001).

#### Relative Abundance of Tubulin Isotypes in *C. elegans* Neurons

The combinatorial tubulin code is controlled by the relative abundance of the multiple isotypes in each cell. scRNA-seq data provides a window to quantitatively assess the isotype composition in a cell at the transcript level (Taylor et al., 2021). In the ciliated neurons, such as the IL2 neurons, the ciliary tubulin *tba-6* showed much higher level of transcription than the ubiquitously expressed *tba-1* and *tba-2*, since *tba-6* counts for 74 and 62% of all α-tubulin transcripts in IL2_DV and IL2_LR neurons, respectively (Figure 2). Thus, in the IL2 neurons, the axonemal microtubules may be mostly made of the ciliary isotype TBA-6. In other cases (e.g., *tba-5* and *tbb-4*), the ciliary tubulins are transcribed at a level comparable or lower than the ubiquitous tubulin isotypes, indicating that the cilia-specific isotypes may not be the major isotype in the axonemal microtubules.

Another example is the mechanosensory tubulins, *mec-12* and *mec-7*, which count for over 99% of the total α- and β-tubulin transcripts, respectively, in the TRNs according to the scRNA-seq data (Taylor et al., 2021). Lockhead et al. (2016) also independently quantified the tubulin transcripts in the PLM neurons (the posterior TRN subtype) using single-neuron RNA-seq and found that *mec-12* and *mec-7* count for 95 and 83% of the overall α- and β-tubulin transcripts, respectively. Both transcriptomic studies suggest that *mec-12* and *mec-7* are the dominating tubulin isotypes in the TRNs, while *tba-1*, *tba-2*, *tbb-1*, and *tbb-2* have very low abundance. Thus, it is reasonable to expect that the 15-protofilament microtubules are mostly made of MEC-12/MEC-7 heterodimers, although this idea has yet to be confirmed with *in vivo* evidence.

However, recent studies using N-terminal GFP knock-in at the endogenous tubulin genes found that GFP::TBA-1 and GFP::TBB-2 showed higher levels of fluorescence than GFP::MEC-12 and GFP::MEC-7 in the TRNs, respectively (Nishida et al., 2021). The authors attributed this discrepancy to different translational efficiencies of the isotypes, but it could also be an artefact of the GFP fusion, since the N-terminal GFP may interfere with tubulin autoregulation that relies on the first four residues (MREI) (Lin et al., 2020). Another discrepancy exists between the broad abundance of *mec-12* in all neurons in the scRNA-seq dataset (Figure 2) and the weak and restricted fluorescence of GFP::MEC-12 in the animal (Nishida et al., 2021). Further studies combining transcriptomic and proteomic approaches will be needed to address such discrepancies.

**FIGURE 3:**
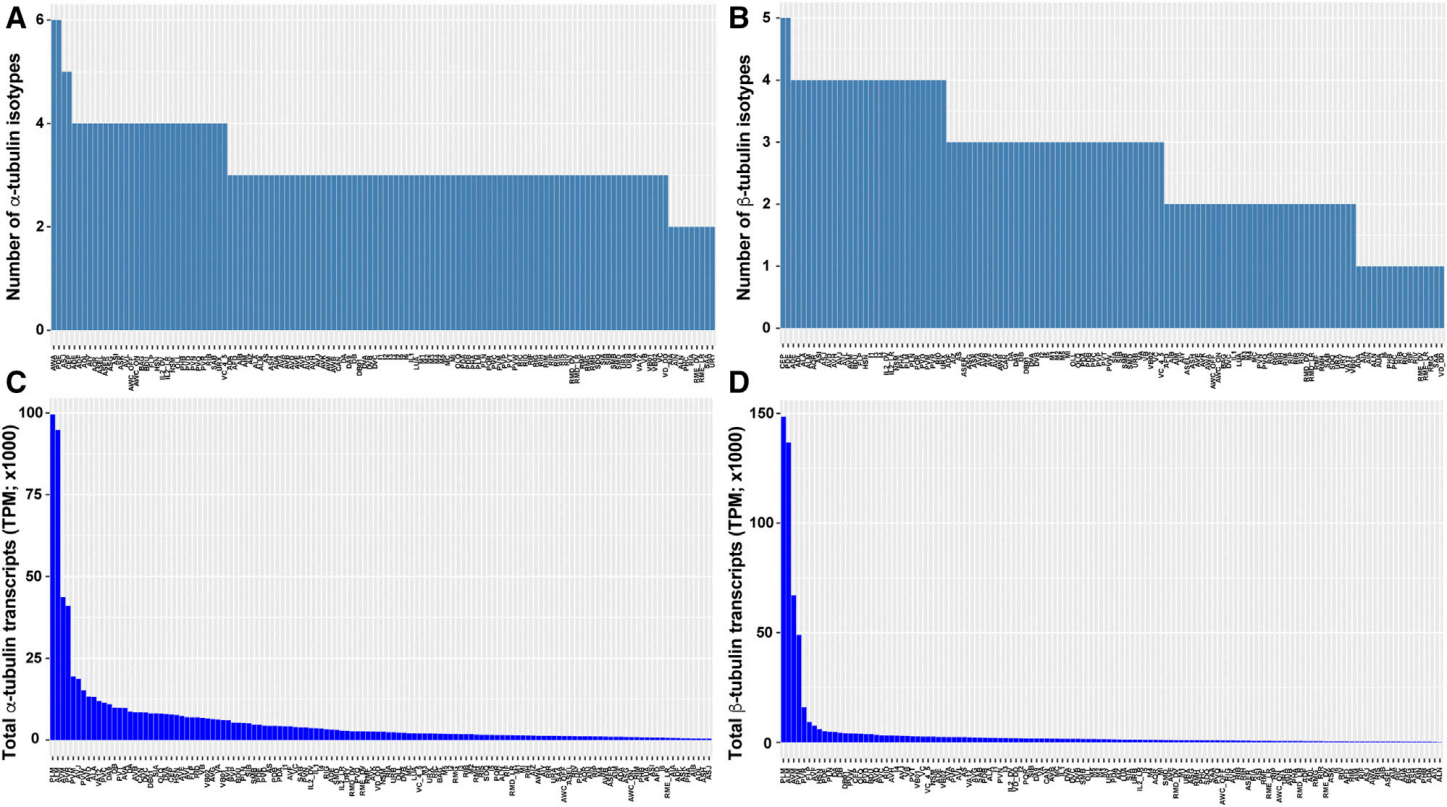
Tubulin isotype numbers and total tubulin transcripts among different neuron types in *C. elegans*. The number of tubulin isotypes expressed (TPM >0) in each neuron type were counted for α-tubulin **(A)** and β-tubulin **(B)** isotypes from the scRNA-seq data. The total number of α-tubulin **(C)** and β-tubulin **(D)** transcripts in each neuron type were calculated by summing the TPM of each isotype.

scRNA-seq data also revealed divergence in the number of isotypes expressed and the total tubulin transcripts among cell types. For example, *C. elegans* neurons on average express three α-tubulin and three β-tubulin isotypes, but significant inequality in the number of isotypes are found among the neuron types (e.g., PDE neurons express 6 α- and 5 β-tubulin isotypes, whereas ALN neurons express 2 α- and 1 β-tubulin isotypes; Figures 3A,B). The total number of tubulin transcripts show even greater variation in the nervous system. Compared to other neurons, the TRNs (especially ALM and PLM neurons) have extraordinarily high level of transcripts for both α- and β-tubulin due to the strong expression of the mechanosensory tubulins, *mec-12* and *mec-7* (Figures 3C,D). This high abundance of tubulin is correlated with the large number of microtubules found in the TRNs. A cross-section of TRN neurite contains ∼31 15-protofilament microtubules which form a bundle, whereas most other neurons have only a few (∼5 per cross-section) 11-profilament microtubules (Chalfie and Thomson 1979). Moreover, deletion of either *mec-12* and *mec-7* leads to significant loss of microtubules (Zheng et al., 2017), suggesting that the number of microtubules in a cell may partly depend on the level of tubulin expression. Strikingly, there is a 245- and 1268-fold difference in total α- and β-tubulin transcripts levels, respectively, between the neurons with the highest and lowest tubulin expression levels (Figures 3C,D). This finding may reflect different needs for microtubules among distinct cell types.

### Functions of Tubulin Isotypes

#### Functional Redundancy and Specificity of Tubulin Isotypes in Embryogenesis

The expression of multiple tubulin isotypes and their high sequence identity suggest that the isotypes may be functionally redundant, while the distinct expression patterns, relative abundance, and subtle sequence difference indicate that each isotype may carry specific functions. This coexistence of redundancy and specificity have been well supported by experimental data in *C. elegans*.

Cell division in the germline and early embryos of *C. elegans* relies heavily on two pairs of tubulins (*tba-1*, *tba-2*, *tbb-1*, *tbb-2*) that assemble into spindle microtubules during mitosis and meiosis. The two α- and two β-tubulin isotypes are mostly redundant. The loss of either TBA-1 or TBA-2 by gene deletion and RNAi caused little viability defects, while simultaneously depleting both is lethal due to the disruption of spindle formation in embryogenesis (Phillips et al., 2004). Similarly, *tbb-1* and *tbb-2* are partially redundant for embryonic viability (Lu et al., 2004), although the null mutation of *tbb-2* caused partial lethality (Ellis et al., 2004; Lu et al., 2004). Missense mutation in *tbb-2* can also cause severe lethality by acting as gain-of-functions to disrupt spindle microtubule assembly in the embryos (Wright and Hunter 2003; Ellis et al., 2004). As the only γ-tubulin isotype, *tbg-1* is found to be required for the nucleation and organization of centrosomal microtubule asters and the positioning dynamics of the spindle (Strome et al., 2001; Hannak et al., 2002).

**FIGURE 4:**
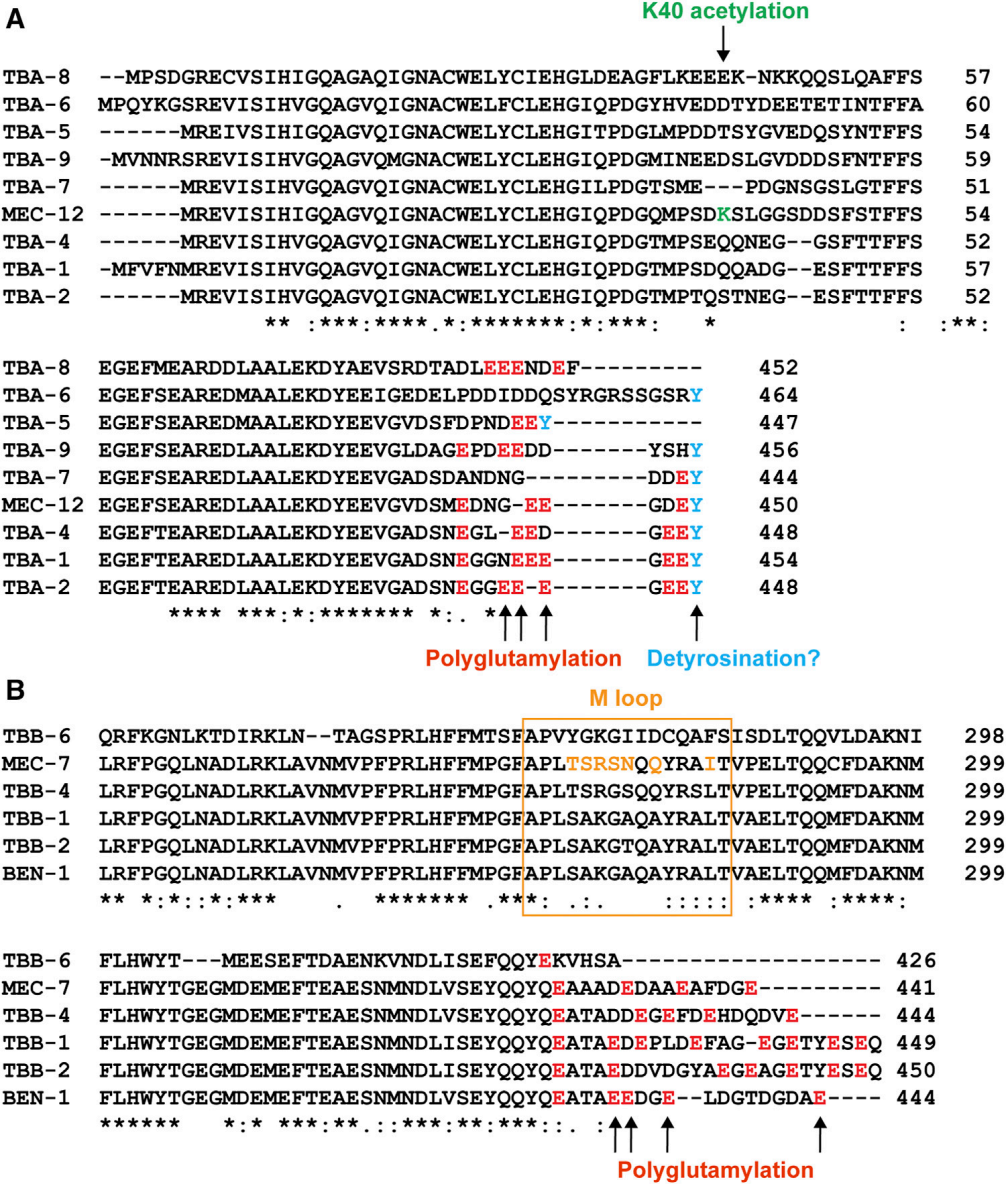
Sequence variation among the tubulin isotypes in *C. elegans*. Alignment of part of the amino acid sequences of α-tubulin **(A)** and β-tubulin **(B)** isotypes. Sites for potential PTMs were color-coded. Green for K40 acetylation, red for polyglutamylation, and blue for potential detyrosination. The sequence for the M loop is labeled by the orange box and the amino acids in the M loop of *MEC-7* that differ from the ubiquitous *TBB-1* and *TBB-2* are in orange as well.

In addition to the redundancy, functional difference also exists between the isotypes. For example, microtubule-severing enzyme katanin prefers TBB-2 over TBB-1 during meiosis since the loss of *tbb-2* leads to severe lethality in activity-weakened katanin mutants, but the loss of *tbb-1* does not cause similar synthetic lethality, suggesting that katanin severs microtubules that contain TBB-2 much more efficiently to support the formation of meiotic spindles (Lu et al., 2004). This different susceptibility to katanin activity may be attributed to the significant sequence divergence of the two isotypes at the C-terminal tail, which is an essential site for the interactions between microtubules and MAPs (Figure 4). Using similar methods, TBA-2 was later found to be preferred over TBA-1 by katanin (Lu and Mains 2005).

In an elegant gene replacement experiment, Honda et al. (2017) found that when the coding exons of the *tbb-1* locus was endogenously edited to encode TBB-2 (the sequences of TBB-1 and TBB-2 only different in 12 amino acids) or vice versa, the resulted embryos expressing two copies of *tbb-1* or *tbb-2* were viable and had similar levels of total β-tubulin expression as the wild-type. Interestingly, the authors also found that the deletion of *tbb-2* reduced the total level of β-tubulin to 19% of the wild-type level and attributed the partial lethality in *tbb-2(-)* mutants to the paucity of total β-tubulin. These results support that TBB-1 and TBB-2 can functionally replace each other for development. However, the two isotypes can also confer distinct dynamic properties of microtubules. For example, the duration of growth for the astral microtubules is longer in embryos expressing only TBB-2 than the ones expressing only TBB-1, while microtubule disassembly occurs more frequently in the TBB-1-only embryos. Moreover, TBA-1 and TBA-2 also appear to differently affect microtubule dynamics, as microtubules composed of TBA-1/TBB-1 had higher growth rate than microtubules made of TBA-2/TBB-1 (Honda et al., 2017). These results support both the redundancy and functional specificity of tubulin isotypes.

#### Ciliary Tubulins Affect the Structure and Function of Cilia

Ciliary tubulins (*tba-5*, *tba-6*, *tba-9*, and *tbb-4*) are found to regulate the structure of the axonemal microtubule and the formation of the cilia in ciliated neurons. For example, missense gain-of-function mutations in *tba-5* and *tbb-4* result in the loss of the distal segments of the cilia in many sensory neurons and destabilize singlet microtubules in cilia, but their deletion alleles do not cause any defects (Hao et al., 2011). So, these two ciliary tubulin isotypes are likely functional redundant with the ubiquitous isotypes also expressed in the neurons. Interestingly, TBA-5 and TBB-4 showed different localization patterns within the cilia. TBB-4 localizes along the length of the cilium but is not found at the transition zone or in the dendrites, whereas TBA-5 localizes in dendrites, the transition zone, and the cilia with enrichment in the distal segment of the cilia (Hao et al., 2011).

The loss-of-function mutation in *tba-6* alters the ciliary ultrastructure in male-specific CEM neurons, causing abnormal curvatures of the axoneme and reduced number of B-tubule singlets. The loss of *tba-6* also affects the intraflagellar transport and extracellular vesicle release in the CEM neurons (Silva et al., 2017). *tba-6*, *tba-9*, and *tbb-4* are also expressed in the male tail sensory ray neurons and their deletions all lead to male mating defects likely because the ray neuron function is compromised (Hurd et al., 2010). Interestingly, the loss of *tbb-4* reduces the accumulation of TBA-6 and TBA-9 in the ray neuron cilia, and the loss of *tba-6* reduces the localization of TBB-4, suggesting that these ciliary isotypes may need to work together to form the specialized ciliary microtubules.

#### Mutations in the Mechanosensory Tubulins Cause Neurite Growth Defects

**FIGURE 5:**
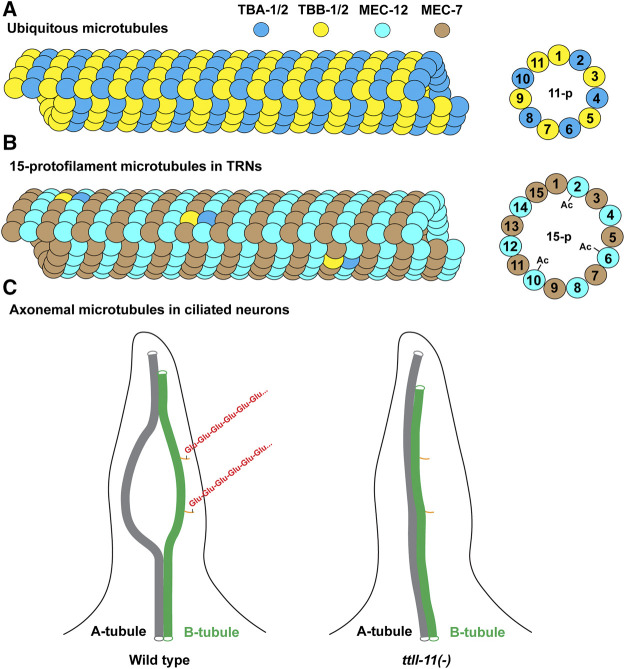
Illustration of microtubule structures in *C. elegans*. **(A)** Most cells in *C. elegans* contain the 11-protofilament microtubules made mostly of the ubiquitous tubulin isotypes TBA-1, TBA-2, TBB-1, and TBB-2. Other expressed isotypes can also be incorporated into the microtubules (not depicted). **(B)** The 15-protofilament microtubules in the TRNs are likely made of MEC-12/MEC-7 heterodimers given their high abundance. The lowly expressed TBA-1/2 and TBB-1/2 may also be incorporated at low levels. Only the microtubules incorporating MEC-12 can be acetylated at the K40 residues. **(C)** The structure of the axonemal microtubules (only one of the nine A-B doublets is depicted). The A-B doublet splay into A-tubule and B-tubule singlets in the middle segment of the cilia. Polyglutmaylation (a chain of “Glu” in red) occurs mostly on the C-terminal tail of the tubulins in the B-tubule. A-B doublet fails to splay into singlets in ttll-11(-) mutants. The orange sticks represent the C-terminal tails of the tubulins on B-tubule. Not all tubulin C-terminal tails on the microtubules are depicted.

The α-tubulin *mec-12* and β-tubulin *mec-7* are highly expressed in the mechanosensory TRNs, which contain many (∼31 per cross-section) large-diameter, 15-protofilament microtubules (Figures 5A,B). In fact, these neurons were originally identified in the electron micrographs by their prominent microtubule structures (e.g., the neuron name “ALM” stands for anterior lateral microtubule) (Chalfie and Thomson 1979). Earlier studies found that missense mutations in either *mec-12* or *mec-7* resulted in the loss of the 15-protofilament microtubules (Savage et al., 1989; Fukushige et al., 1999; Bounoutas et al., 2009). Recent studies using the deletion alleles, however, found that only the *mec-7(-)* mutants but not the *mec-12(-)* mutants lost the 15-protofilament structure (Zheng et al., 2017). *mec-7(-)* mutants instead have the small-diameter 11-protofilament microtubules. Both *mec-7(-)* and *mec-12(-)* mutants have significantly fewer (∼6 or 7 per cross-section) microtubules compared to the wild type. This result suggests that the role of MEC-7 in producing the 15-protofilament microtubules cannot be replaced by other β-tubulin isotypes (e.g., TBB-1 and TBB-2) in the TRNs, whereas MEC-12 can be replaced by other α-tubulin isotypes. MEC-7 may possess unique properties that modulate the protofilament number and microtubule organization (e.g., unique sequences in the M loop that affects lateral contact; Figure 4B). The reduced number of microtubules in *mec-12(-) *and *mec-7(-)* mutants is likely caused by the decrease in total tubulin levels, since *mec-12* and *mec-7* count for >99% of the total α- and β-tubulin transcripts, respectively, according to the scRNA-seq data. Although the removal of one tubulin isotype may cause the upregulation of other isotypes (e.g., TBB-1 proteins are upregulated ∼3-fold in *tbb-2(-)* mutants (Ellis et al., 2004)), it is unclear whether the upregulation of other isotypes can entirely compensate for the loss of the highly transcribed *mec-12* and *mec-7* in the TRNs. Moreover, the abundance of microtubules appeared to be essential for mechanosensation in TRNs, since both *mec-12(-)* and *mec-7(-)* mutants showed significantly reduced touch sensitivity.

TRNs extend long antero-posteriorly running neurites that mediate the sensing of mechanical stimuli along the body. Deletion of *mec-12* or any of the four ubiquitous tubulin isotypes individually did not produce any obvious defects on neurite growth, and the deletion of *mec-7* only caused mild defects (Lockhead et al., 2016; Zheng et al., 2017). These results suggest that the reduced number of microtubules and the 11-protofilament microtubules are sufficient for supporting general neurite growth in the TRNs. Thus, the tubulin isotypes appeared to be generally redundant in regulating neuronal morphogenesis.

Through extensive genetic screens, a large collection of *mec-12* and *mec-7* missense mutations (99 alleles in total) had been isolated and characterized for their effects on neurite growth (Zheng et al., 2017; Lee et al., 2021). In addition to many loss-of-function mutants, two distinct types of gain-of-function alleles were found among the missense alleles, and they had much stronger effects on neurite growth than the deletion alleles. The antimorphic gain-of-function mutations were dominant or semi-dominant and caused severe shortening of all TRN neurites. The mutated residues mostly locate at the GTP binding site and the intradimer and interdimer interfaces, suggesting that the antimorphic mutations may cause the formation of poisonous tubulin dimers, whose incorporation could terminate microtubule polymerization and induce instability, thus blocking neurite growth. On the other hand, the neomorphic gain-of-function mutations resulted in hyperstable microtubules and ectopic neurite growth; these mutations were mostly mapped to the exterior surface of the microtubules and may affect the interaction with MAPs (Zheng et al., 2017). In genetic terms, antimorphs are dominant-negative mutations that act in opposition to normal gene activity, and neomorphs cause gain of gene function that is different from the normal gene function.

Such structure-function analysis, which were enabled by the phenotypical characterization of a large number of tubulin missense alleles in *C. elegans* TRNs, is valuable in light of the discovery of over 100 tubulin mutations associated with neurodevelopmental disorders in humans (Tischfield et al., 2011; Fourel and Boscheron 2020). In fact, previous work has shown that *C. elegans* TRNs can be used to evaluate the cellular impact of disease-associated human tubulin mutations by engineering these mutations into the endogenous *mec-12* and *mec-7* loci and then examining the phenotype on neurite growth patterns (Zheng et al., 2017). Given the ease of gene editing in *C. elegans*, such phenotypical evaluation can be done in less than two weeks, which provides a tool to understand the nature of the human tubulin mutations.

Lastly, studies of the *mec-12* and *mec-7* alleles also found complex genetic interactions between the tubulin mutations, including epistatic, additive, synthetic, and balancing effects (Lee et al., 2021). For example, the effects of neomorphic gain-of-function mutants can be suppressed by loss-of-function and antimorphic gain-of-function mutations. Neomorphic alleles often show additive effects as expected, but sometimes a weaker neomorphic allele could also mask the effects of a stronger neomorphic allele in inducing ectopic neurite growth. Moreover, the microtubule-stabilizing and -destabilizing effects of neomorphic and antimorphic alleles were found in some cases to neutralize each other to allow normal neurite growth pattern in the double mutants (Lee et al., 2021). These results are in line with earlier molecular genetics studies on tubulins in other organisms (Morris et al., 1979) and can help understand how tubulin isotypes work together.

#### Other Functional Studies of Tubulins

Since microtubules serve as the tracks for intracellular transport, mutations in tubulins also disrupt the trafficking of organelles and vesicles. For example, mutations in *mec-12* and *mec-7* cause abnormalities in the transport of synaptic vesicles and mechanosensitive channel protein MEC-2 in the TRNs (Huang et al., 1995; Zheng et al., 2017).

Outside of the TRNs, missense mutations are also found to affect neuronal development. For example, a gain-of-function missense mutation in *tba-1* causes defects in axonal extension and neuromuscular synapse formation in the DD motor neurons, which leads to altered locomotion, although *tba-1(-)* null mutants does not show any of these defects (Baran et al., 2010). Interestingly, *tbb-2(-)* null mutants also show axonal growth defects in DD neurons and reduced synapse numbers, suggesting that the missense mutation in *tba-1* may mostly affect microtubules formed by TBA-1 and TBB-2. The same *tba-1* missense mutation delays synapse remodeling in DD neurons, and this delay is exacerbated by the loss of the MAPKKK DLK-1 (Kurup et al., 2015). Further studies found that the *tba-1* gain-of-function mutation and the *dlk-1(-)* null mutation have synergistic effects in reducing microtubule dynamics, which leads to defects in synaptic remodeling.

Both deletion and missense mutations in *ben-1* (or *tbb-5*) confer resistance to the microtubule-depolymerizing drug benomyl in a dominant fashion, suggesting that *ben-1* is haploid insufficient (Driscoll et al., 1989). Except for the resistance to the neurological phenotypes induced by benomyl, *ben-1(-)* mutants had no other obvious defects. Although *ben-1* was thought, for a long time, to be the only tubulin isotype sensitive to benomyl, which may result from specific features in the sequence of BEN-1, a later study isolated a rare *tbb-2* missense mutation that also confers benomyl resistance (Wright and Hunter 2003).

Tubulins were identified in genome-wide RNAi screens for genes required in cell migration. For example, *tba-1*, *tba-2*, *tba-4*, *tbb-1*, *tbb-2*, and *tbg-1* are all found in a gene network that regulates the migration of distal tip cells during the development *C. elegans* gonad (Cram et al., 2006). It is worth noting that both a missense and a nonsense mutation in *tba-7* were previously found to cause ectopic neurite growth and enhanced axonal regeneration in the TRNs (Zheng et al., 2017; Kim et al., 2018). However, follow-up studies found that these defects should be attributed to two independent background mutations in the microtubule-depolymerizing kinesin-13 *klp-7*, which is closely linked to *tba-7* (Lu and Zheng 2021). The function of *tba-7* and other tubulins (including *tbb-6* and *tba-8*) awaits further investigations.

### The Effects of Tubulin PTMs in *C. elegans*

Tubulins undergo a variety of posttranslational modifications (PTMs) that regulate the microtubule structure, dynamics, and association with MAPs. Functional PTMs include α-tubulin K40 and K252 acetylation, detyrosination, and Δ2-modification and β-tubulin Q15 polyamination and S172 phosphorylation, as well as polyglutamylation and polyglycylation of the C-terminal tails of both α- and β-tubulin isotypes; their effects in other organisms have been extensively reviewed elsewhere (Magiera and Janke, 2014; Janke and Magiera, 2020). We will focus on the reported function of tubulin PTMs in *C. elegans*.

#### MEC-12 K40 Acetylation

Among the nine α-tubulin isotypes in *C. elegans*, only MEC-12 possess the lysine 40 residue (Figure 4A). So, only MEC-12 is subjected to K40 acetylation, which is catalyzed redundantly by two α-tubulin acetyltransferases—MEC-17 and ATAT-2 (Akella et al., 2010). The loss of either one alone did not reduce MEC-12 acetylation levels, but acetylation is not detectable in the *mec-17(-)* *atat-2(-)* double mutants (Topalidou et al., 2012). Although K40 acetylation has been found in long-lived microtubules (Schulze et al., 1987), exactly how it contributes to microtubule stability remains unclear. As the only PTM site located inside the lumen of microtubules (Figure 5B), acetylated K40 is found by recent studies to reduce inter-protofilament interactions and thus prevent long-lived microtubules from breaking under mechanical stresses (Portran et al., 2017; Eshun-Wilson et al., 2019). However, it remains debatable whether microtubules become stable after acetylation or whether long-lived microtubules accumulate acetylation (Palazzo et al., 2003).

In *C. elegans*, MEC-17 is specifically expressed in the six TRNs, whereas ATAT-2 is broadly expressed in the nervous system. The deletion of MEC-17 reduces the number and length of microtubules and converts the 15-protofilament organization to 13-protofilament in the TRNs (Cueva et al., 2012; Topalidou et al., 2012). These changes in microtubule structure are not likely caused by the loss of K40 acetylation because MEC-12 K40 acetylation level in *mec-17(-)* mutants is comparable to the wild-type animals due to the existence of ATAT-2. Disorganized microtubules in *mec-17(-)* mutants lead to reduced touch sensitivity and abnormal neurite morphology, as well as early-onset axonal degeneration (Topalidou et al., 2012; Neumann and Hilliard, 2014). Many of these defects, however, can be rescued by the expression of enzymatically inactive MEC-17, which further supports that at least part of MEC-17’s function is independent of acetylating MEC-12 K40. Exactly how MEC-17 exerts these functions remains to be understood.

On the other hand, some studies attempted to directly assess the effect of K40 in *mec-12* by transgenic expression of the acetyl-mimic K40Q and the nonacetylatable K40R mutants. Akella et al. (2010) found that expression of either K40Q or K40R could partially rescue touch sensitivity in *mec-12(e1620)* mutants to the same level but neither could rescue as well as the *mec-12(+)* transgene. Cueva et al. (2012) found that *mec-12(K40R)* transgene could not restore the number and length of microtubules and the protofilament number in *mec-12(e1620)* mutants to the same level as the *mec-12(+)* transgene could. These results are difficult to interpret because 1) the *mec-12(e1620)* allele used is in fact not a null allele but an antimorphic gain-of-function (Zheng et al., 2017); 2) transgenes often have variable expression levels and the abundance of tubulins would affect their functions. Better experimental design (e.g., editing the endogenous *mec-12* locus) will be needed to address the role of MEC-12 K40 acetylation.

#### Tubulin Polyglutamylation

The C-terminal tails of tubulins contain approximately 12 amino acids, which are subjected to at least five PTMs (Janke and Magiera, 2020). One such PTM is polyglutamylation, which adds multiple glutamates to the γ-carboxyl group of any of the glutamic acid residues in the C-terminal tail (Edde et al., 1990). Tubulin polyglutamylation were catalyzed by enzymes of the tubulin tyrosine ligase-like (TTLL) family and the glutamate side chain can also be removed by the cytosolic carboxypeptidases (CCPs) in a process known as deglutamylation (Janke et al., 2005; Rogowski et al., 2010). Mammalian genomes encode multiple TTLL and CCP proteins (e.g., mice have at least nine TTLLs and six CCPs), which showed preferences towards either α- or β-tubulins; some TTLLs are responsible for initiating the glutamylation, and others for elongating the glutamate sidechain (van Dijk et al., 2007; Rogowski et al., 2010). Tubulin polyglutamylation is known to regulate axonemal motility by modulating the activity of dynein motors in the cilia (Kubo et al., 2010; Suryavanshi et al., 2010) and regulate microtubule stabilities in neurons by stimulating spastin-mediated severing of microtubules (Lacroix et al., 2010).

The *C. elegans* genome encodes six TTLLs (TTLL-4, -5, -9, -11, -12, and -15) and two CCPs (CCPP-1 and CCPP-6), although TTLL-12 is the ortholog of mammalian TTLL12, which lacks glutamylase and glycylase activity (Yu et al., 2015). Biochemical studies found that TTLL-4 and CCPP-6 are the major tubulin polyglutamylase and deglutamylase, respectively, in ciliated sensory neurons, because either removing *ttll-4* or overexpressing CCPP-6 abolished the polyglutamylation signal in immunofluorescence (Kimura et al., 2010). In the male-specific ciliated neurons, CCPP-1 regulates axonemal microtubule structures and the ciliary localization of kinesin-3 KLP-6 and its cargo polycystin PKD-2 (O'Hagan et al., 2011). The loss of *ccpp-1* results in B-tubule defects, microtubule disorganization, and ciliary fragmentation, which is consistent with the findings that B-tubules are the main site of polyglutamylation (Kubo et al., 2010). At the behavioral level, in addition to mate-sensing defects, *ccpp-1(-)* mutants showed a progressive, age-dependent defects in dye-filling (uptake of fluorescent dye by sensory cilia) and osmotic avoidance, indicating that hyperpolyglutamylation may disrupt ciliary maintenance (O'Hagan et al., 2011).

Further studies found that TTLL-11 is required for ciliary microtubule polyglutamylation in males (O'Hagan et al., 2017). Intriguingly, *ttll-11(-)* mutants showed some similar defects as *ccpp-1(-)* mutants, including the abnormal enrichment of PKD-2 in the cilia and defects in extracellular vesicle release from the ciliary base, suggesting that an optimal level of glutamylation is needed for ciliary functions. The loss of TTLL-11 alters the axonemal microtubule architecture by preventing the splaying of the A-B doublets into A- and B-tubule singlets (Figure 5C); and *ccpp-1(-)* *ttll-11(-)* double mutants showed similar defects as the *ttll-11(-)* single mutants (O'Hagan et al., 2017). These results suggest that polyglutamylation regulates microtubule organization in the cilia. It is worth noting that *tba-6(-)* mutants also showed similar defects in splaying the A-B tubules (Silva et al., 2017), but the C-terminal tail of TBA-6 does not seem to contain residues for polyglutamylation (Figure 4A). One possible explanation is that TBA-6 functions together with other tubulin isotypes, such as TBB-4, which has polyglutamylation sites. The loss of TBA-6 may affect TBB-4 incorporation into the axonemal microtubules and thus reduce the overall glutamylation levels of the microtubules. In fact, Hurd et al. (2010) has previously observed reduced TBB-4 localization in the cilia of *tba-6(-)* mutants, supporting this hypothesis. Whether polyglutamylation of TBB-4 indeed regulates the organization of ciliary microtubules awaits further investigation.

Moreover, mutations in *ttll-11* also suppressed the abnormal localization and increased velocity of kinesin motors KLP-6 and OSM-3 in *ccpp-1(-)* mutants, suggesting that tubulin polyglutamylation serves as an important regulator of kinesin motility in intraflagellar transport (O'Hagan et al., 2017). In a separate study, Chawla et al. (2016) found that although the loss of *ttll* genes individually did not affect male mating efficiency, the *ttll-4(-) ttll-5(-) ttll-11(-)* triple mutants showed defects in the response step of male mating. Thus, it appears both hyperglutamylation and hypoglutamylation disrupt the function of male-specific cilia. Nevertheless, the triple mutants did not show defects in embryonic viability, brood size, dye-filling, and osmotic avoidance (Chawla et al., 2016).

Excessive amount of polyglutamylation causes neurodegeneration in mammals (Magiera et al., 2018). In *C. elegans* amphid and phasmid neurons, the loss of CCPP-1 leads to progressive ciliary degeneration, which results in dye-filling defects in adults but not at early larval stages. Mutations in *ttll-4*, *ttll-5*, and *ttll-11* individually could all at least partially suppress the age-dependent dye-filling defects of *ccpp-1(-)* mutants (Power et al., 2020), supporting that polyglutamylation regulates ciliary stability and hyperglutamylation promotes ciliary microtubule degeneration.

In the non-ciliated TRNs, hyperglutamylation alters microtubule dynamics and blocks axonal regeneration. Loss of *ccpp-6* reduced the length of PLM regrowth following laser axotomy, whereas the regrowth is enhanced in *ttll-5(-)* mutants (Ghosh-Roy et al., 2012). Microtubules in *ccpp-6(-)* mutants are not able to sustain stabilized growth, while microtubules in *ttll-5(-)* mutants transition into persistent growth faster than the wild-type after axotomy. The same study also suggested that polyglutamylated microtubules may have increased sensitivity to the microtubule-destabilizing kinesin-13 KLP-7, supporting the idea that polyglutamylation may reduce microtubule stability in non-ciliated neurons.

#### Other Tubulin Posttranslational Modifications

Apart from K40 acetylation and polyglutamylation, the existence and function of other tubulin PTMs in *C. elegans* were unclear. For example, polyglycylation also occurs at the C-terminal tail of tubulins by adding glycine side chains to the same glutamate residues that are subjected to polyglutamylation (Janke and Magiera 2020). Polyglycylation appears to mostly label the axonemal microtubules in motile flagella and cilia. *C. elegans* has neither motile cilia nor orthologs of mammalian polyglycylase, and immunostaining against polyglycylation shows no signal (Kimura et al., 2010).

The C-terminal tail of α-tubulin could also undergo detyrosination, which removes the terminal tyrosine residue. Detyrosination can lead to the removal of the exposed penultimate glutamate residue, generating Δ2-tubulin. Both detyrosination and Δ2-modificatiosn are associated with long-lived stable microtubules and the tyrosination/detyrosination state modulate the interaction with plus end-binding proteins (Peris et al., 2006), the MT-depolymerization activity of kinesin-13 (Peris et al., 2009), and the motility of dynein-dynactin motor (McKenney et al., 2016). Detyrosination is mediated by the tubulin carboxypeptidases, vasohibins (VASH1/VASH2), and their stabilizing chaperone SVBP (Nieuwenhuis et al., 2017). Most *C. elegans* α-tubulin isotypes contain the terminal tyrosine (Figure 4A), but *C. elegans* has no ortholog of vasohibins, raising the question whether detyrosination occurs in *C. elegans*. Mutating the terminal tyrosine to alanine in TBA-1 and TBA-2 abolishes tyrosination signal and causes defects in the centration and rotation of centrosome in early embryos, similar to the defects found in dynactin *dnc-1* mutants, which supports that detyrosination may regulate the interaction with dynein-dynactin complex (Barbosa et al., 2017). However, this study only indicates the importance of the terminal tyrosine but could not serve as evidence for detyrosination in *C. elegans*. In addition, detyrosinated tubulins can be retyrosinated by tubulin tyrosine ligase (TTL) (Nieuwenhuis and Brummelkamp 2019). *C. elegans* does not seem to have a direct homolog of mammalian TTL, and whether any of the six TTLL enzymes in *C. elegans* can mediate tubulin retyrosination remains unknown.

### Future Directions

The existence of multiple tubulin isotypes with distinct expression patterns and specialized functions, together with the ever-expanding variety of tubulin PTMs, lead to the fascinating concept of “tubulin code”. The use of a simple organism such as *C. elegans* can be instrumental for cracking the tubulin code and understanding the regulation of microtubule structure and function by tubulin isotype composition and PTMs. Since CRISPR/Cas9-mediated gene editing is extremely easy and robust in *C. elegans*, we expect genetic studies to continue providing important insights into the functional specificity of tubulin isotypes and the effects of tubulin PTMs. Below, we list some future research directions.

The function of each tubulin isotype can be elucidated by analyzing the phenotype of individual tubulin deletion mutants guided by the tubulin expression map. Specific combination of tubulin mutants could also be constructed based on their expression patterns to overcome the expected genetic redundancies. This systematic analysis will provide a full picture of the expression and functional diversity of tubulin isotypes at the whole-genome and whole-organism level. To assess isotype-specific properties, gene replacement studies can be conducted to convert the endogenous locus of one tubulin isotype to another as done by Honda et al. (2017). This type of gene editing experiments can also be used to address whether the relative abundance of the tubulin isotypes play a role in defining microtubule properties. For example, the *mec-12* and *tba-1* coding regions in the genome can be swapped without changing their promoters and then the microtubule properties can be analyzed to test whether the relative abundance of MEC-12 and TBA-1 is important in TRNs.

The study of the tubulin code will also benefit from the development of the recombinant tubulin technology. Previous *in vitro* studies of tubulins and microtubules mostly rely on the use of tubulin proteins isolated from the bovine brain, which is known to contain tubulins with mixed isotypes and unknown PTMs. Recent studies by Ti et al. (2018) have shown the promise of generating isotypically pure microtubules with define PTMs from a recombinant source. Through cryo-EM reconstructions, the authors found that human TUBA1B/TUBB3 heterodimers form microtubules with 13 protofilaments, while TUBA1B/TUBB2 dimers form microtubules with 14 protofilaments, suggesting that β-tubulin isotype can determine protofilament number. This result echoes the findings that MEC-7 is required for the 15-protofilament microtubule structure in the *C. elegans* TRNs. Structural studies of the microtubules made from recombinant MEC-12/MEC-7 dimers and comparison with the microtubules made of the ubiquitous tubulins (e.g., TBA-1/TBB-1) can provide the definitive answer for how a specific isotype determines the 15-profilament structure.

The studies of the effects of tubulin PTMs need to include more precise gene editing experiments. Previous studies on tubulin PTMs may be limited in two aspects. First, the effects of the PTMs were studied by mostly deleting the enzymes that add or remove specific PTMs. However, these enzymes may have substrates other than tubulins or have activities independent of its enzymatic functions (e.g., the acetyltransferase MEC-17). So, the effects resulted from losing an enzyme may not be entirely attributed to the change of tubulin PTMs. Second, in some cases, the effects of tubulin PTMs were revealed by the expression of tubulin mutants with PTM-mimicking or unmodifiable amino acid substitutions. Such transgenic expression may create artifacts due to uncontrolled expression levels and potential tubulin mRNA autoregulation (Lin et al., 2020). Therefore, precise engineering of the endogenous tubulin loci to install PTM-mimicking or -inactivating mutations will be better in revealing the role of the PTM. Nonetheless, polymodifications, such as polyglutamylation and polyglycylation, are difficult to mimic genetically. Advances in chemical biology will be needed to create tools for such studies.

In the coming years, we will likely see more and more tubulin PTMs to be identified and their functions to be studied, especially given that proteomic studies have identified 80 residues in tubulins that are subjected to one or multiple types of PTMs (Liu et al., 2015). We expect *C. elegans* to serve as a valuable tool in understanding the role of this network of tubulin PTMs.
